# Molecular and biochemical characterisation of a novel mutation in *POLG *associated with Alpers syndrome

**DOI:** 10.1186/1471-2377-11-4

**Published:** 2011-01-14

**Authors:** André Schaller, Dagmar Hahn, Christopher B Jackson, Ilse Kern, Christophe Chardot, Dominique C Belli, Sabina Gallati, Jean-Marc Nuoffer

**Affiliations:** 1 Division of Human Genetics, University Hospital Bern, Bern, Switzerland; 2 Institute of Clinical Chemistry, University Hospital Bern, Bern, Switzerland; 3 Department of Paediatrics, University of Geneva Children's Hospital, Geneva, Switzerland; 4 Paediatric Surgery Unit, University of Geneva Children's Hospital, Geneva, Switzerland; 5 Paediatric Surgery Unit, Hôpital Necker-Enfants malades, Paris, France

## Abstract

**Background:**

DNA polymerase γ (*POLG*) is the only known mitochondrial DNA (mtDNA) polymerase. It mediates mtDNA replication and base excision repair. Mutations in the *POLG *gene lead to reduction of functional mtDNA (mtDNA depletion and/or deletions) and are therefore predicted to result in defective oxidative phosphorylation (OXPHOS). Many mutations map to the polymerase and exonuclease domains of the enzyme and produce a broad clinical spectrum. The most frequent mutation p.A467T is localised in the linker region between these domains. In compound heterozygote patients the p.A467T mutation has been described to be associated amongst others with fatal childhood encephalopathy. These patients have a poorer survival rate compared to homozygotes.

**Methods:**

mtDNA content in various tissues (fibroblasts, muscle and liver) was quantified using quantitative PCR (qPCR). OXPHOS activities in the same tissues were assessed using spectrophotometric methods and catalytic stain of BN-PAGE.

**Results:**

We characterise a novel splice site mutation in *POLG *found *in trans *with the p.A467T mutation in a 3.5 years old boy with valproic acid induced acute liver failure (Alpers-Huttenlocher syndrome). These mutations result in a tissue specific depletion of the mtDNA which correlates with the OXPHOS-activities.

**Conclusions:**

mtDNA depletion can be expressed in a high tissue-specific manner and confirms the need to analyse primary tissue. Furthermore*, POLG *analysis optimises clinical management in the early stages of disease and reinforces the need for its evaluation before starting valproic acid treatment.

## Background

Mitochondria have their own small 16.5 kb circular double stranded DNA encoding 22 tRNAs, 2 rRNAs and 13 polypeptides, that are absolutely essential for electron transport and oxidative phosphorylation. The remaining 1000-1500 proteins required for mitochondrial biogenesis are encoded by the nuclear genome and are imported into the mitochondria [1]. These include the proteins involved in mitochondrial DNA (mtDNA) replication, which, if defective, can produce mtDNA mutations leading to mitochondrial dysfunction and disease [2].

Among the 16 DNA polymerases identified in the eukaryotic cell so far, only DNA polymerase γ (pol γ) is known to function in mitochondria [3-5]. The holoenzyme of human pol γ is composed of the catalytic subunit (encoded by *POLG *at chromosomal locus 15q25) and a homodimer of its accessory factor (encoded by *POLG2 *at chromosomal locus 17q24.1) [6]. Mutations in the *POLG *gene have emerged as one of the most common causes of inherited mitochondrial disease in children and adults. They are responsible for a heterogeneous group of at least six major phenotypes of neurodegenerative diseases that include: 1) childhood Myocerebrohepathopathy Spectrum disorders (MCHS), 2) Alpers syndrome [7], 3) Ataxia Neuropathy Spectrum (ANS) disorders [8], 4) Myoclonus Epilepsy Myopathy Sensory Ataxia (MEMSA), 5) autosomal recessive Progressive External Ophthalmoplegia (arPEO), and 6) autosomal dominant Progressive External Ophthalmoplegia (adPEO) [9-11]. As a consequence of POLG failure, accumulation of multiple mtDNA deletions and/or depletion of mtDNA in postmitotic tissues such as muscle, brain and liver is noted [12]. Additionally, various combinations of OXPHOS complex deficiencies have been reported due to POLG mutations [13-17].

Furthermore, recent studies in this area reinforced, in particular, evidence that certain mutations in *POLG *can lead to a range of clinical phenotypes which predispose to development of fatal liver failure after exposure to valproic acid (VPA) [15,18].

In this paper we describe the molecular genetic analysis of *POLG *in a 3.5 years old boy with VPA induced fatal liver failure (Alpers-Huttenlocher syndrome, AHS). The consequences of the findings were further investigated at molecular and biochemical levels.

## Methods

### Patient

A 3 7/12 year old boy, from non-consanguineous parents, with global developmental delay and ataxia was treated with valproate because of focal seizures with secondary generalisation. After 2 months, he developed acute liver failure (INR 29.95, PTT 68,9'',fibrinogen <0.5 g/l, total bilirubin 152 μmol/l, ASAT 169 U/L, ALAT 139 U/L, NH3 124 μmol/l). AHS was diagnosed based on medical history, and medical work-up demonstrated no other causes of acute liver failure. Due to the unavoidable severe progression of the neurological impairment, liver transplantation[19-21] was not proposed and the child died within two days. His parents gave informed consent for genetic studies on the collected samples (blood, skin, muscle and liver biopsies). The study protocol was approved by the local ethic commission of Bern (KEK Nr. 84/02).

### Mutation Analysis and DNA Sequencing

Genomic DNA was extracted from EDTA-stabilised venous blood samples applying the QIAamp DNA kit according to the manufacturer's instructions. All 22 coding exons of the *POLG *were amplified from genomic DNA by means of PCR using primers listed in additional file 1. Mutation analysis of the amplified exons was performed by SSCP as described previously [22]. PCR products were sequenced employing BigDye Terminator Chemistry (Applied Biosystems) and separated on an ABI 3100 DNA Sequencer. Data were analysed with SeqScape version 2.1.1 software (Applied Biosystems).

### Cell culture

Primary fibroblast cultures were established from a skin biopsy and cultured in minimal essential medium (MEM) supplemented with 10% fetal calf serum, 4 mmol/L l-glutamine, 2 μmol/L uridine, 1 μmol/L sodium pyruvate, 50 U/ml penicillin, and 50 μg/ml streptomycin at 37°C and 5% CO_2_.

### Transcript analysis

Primary fibroblasts were grown for 8 h in 75 mM caffeine prior to preparation of RNA in order to minimise nonsense mediated mRNA decay. Total RNA was isolated using the QIAgen RNeasy Kit according to the manufacturer's instructions. Random oligohexamer primed RNA (up to 1 μg) was reverse transcribed in a final volume of 25 μl using the SuperScript II First-Strand cDNA Synthesis System (Invitrogen) according to the manufacturer's recommendations. One-twenty-fifth of single stranded cDNA was used as a template to amplify the fragment spanning exons 5-9 of *POLG *using the following two oligonucleotides: POLGex5 fwd 5'-GCACCATGAAGGACATTCGT-3' and POLGex9 rev 5'-GCCATGACATCTTGTTGAAACT-3'. Cycling conditions using HotStar Taq DNA Polymerase (Qiagen) were 95°C for 15 min, 32 cycles of 15 s at 95°C, 15 s at 58°C and 1 min at 72°C and a final extension step of 5 min at 72°C. RT-PCR products were separated on an agarose gel and extracted for subsequent sequencing.

For determination of splicing efficiency of the c.1251-2A > T allele, cDNA was synthesised using two oligonucleotides spanning from exon 6 to exon 8: POLGex6 fwd 5'-TGTGCCCAGGACGTGTG-3' and POLGex8 rev 5'-CCGAGGTCTTCCTGATCCAT-3' essentially as describe above. The RT-PCR product was subsequently subcloned into pCR2.1-TOPO plasmid using the TOPO-TA Cloining Kit (Invitrogen). Single colonies were picked and inserts were directly amplified using M13 forward and reverse primers and subjected to DNA sequencing as described above.

### mtDNA quantification

Total genomic DNA from muscle, liver and fibroblast specimens were isolated using the QIAamp DNA kit according to the manufacturer's instructions. Mitochondrial DNA content was determined by real-time PCR essentially as described by Bai and Wong [23] using SYBR Green fluorescence dye for detection of amplification. The sequence for reverse primer D-loop was replaced by the following oligonucleotide: 5'-CCGTGAGTGGTTAATAGGGTG-3'. The real-time PCR reactions for each locus (D-loop, tRNALeuUUR, ND4, ATP8 and β2 M) were performed in duplicate in 20 μl reactions containing 10 μl SYBR ^® ^Premix ExTaq (Perfect real time, Takara Bio USA), 0.5 μM of each primer for a corresponding target region and 4 ng of total genomic DNA. Real time PCR conditions were 5 min at 95°, followed by 40 cycles of 30 s at 95°C, 15 s at 60°C and 10 s at 72°C. Fluorescent signal intensity of PCR products was recorded and analysed on a LightCycler 480 instrument (Roche Diagnostics) using LightCycler^® ^480 software. The threshold cycle or C_T _value within the linear exponential phase was used to construct the standard curve and to measure the original copy number of DNA template. Fibroblasts control values were established from patients with circumcisions or auriculoplasty, muscle and liver controls were from biopsies of patients without clinical and biochemical suspicion of a mitochondrial disorder.

### Biochemical assays

Isolation of mitochondria from skin fibroblasts, preparation of skeletal muscle- and liver homogenates (600 g supernatants) were performed as described [24,25]. The activities of the individual respiratory chain complexes and the mitochondrial matrix marker enzyme citrate synthase were measured spectrophotometrically with an UV-1601 spectrophotometer (Shimadzu) using 1 ml sample cuvettes thermostatically maintained at 30°C according to Birch-Machin and Douglas [25]. Values were estimated by the difference in activity levels measured in the presence and absence of specific inhibitors and expressed as ratios to the mitochondrial marker enzyme citrate synthase (mU/mU citrate synthase), which was determined as described [26]. Fibroblasts control values were established from patients with circumcisions or auriculoplasty, muscle and liver controls were from biopsies of patients without clinical and biochemical suspicion of a mitochondrial disorders.

For catalytic in gel staining, 600 g supernatants of muscle- and liver homogenates were centrifuged (30 min, 4°C,13000 rpm) and the oxidative phosphorylation complexes were solubilised by 5 mg digitonin per mg protein before separation by BN-PAGE as recommended [27]. For the first dimension gradient gels of 4.5 - 13% were used. Catalytic staining of strips of the first dimension were performed as reported [28].

## Results

**Figure 1:**
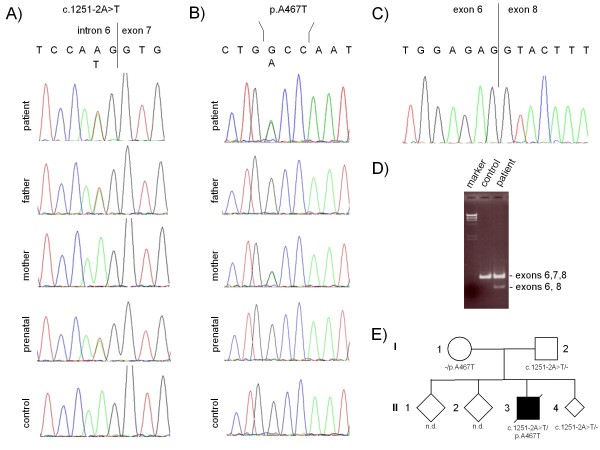
**Molecular genetic analysis of *POLG***. a) Electropherogram showing the presence or absence of the novel splice site mutation in intron 6 of the patient, his parents and the prenatal diagnosis in the gDNA. b) Electropherogram showing the presence or absence of the p.A467T mutation in exon 7 of the patient, his parents and the prenatal diagnosis in the gDNA. c) Electropherogram showing aberrantly spliced POLG mRNA lacking exon 7 by sequencing the gel isolated lower band derived from patient's lane in d). d) Agarose gel showing POLG transcript analysis POLG mRNA derived from fibroblasts. An aberrant splicing product lacking exon 7 is detected in patients cDNA. e) Pedigree of the family including the genotype.

Sequence analysis of patients DNA revealed compound heterozygosity for mutations in *POLG*. Beside the most common *POLG *mutation c.1399G > A/p.A467T (Figure 1A), a novel splice site mutation in intron 6 was identified (c.1251-2A > T) (Figure 1B). The splice site mutation results in exon 7 skipping (Figure 1C,D), but does not affect the reading-frame, hence, the aberrantly spliced POLG transcript should not be vulnerable to undergo nonsense mediated RNA decay (NMD). To assess the degree of exon skipping for the c.1251-2A > T allele PCR products derived from *POLG *cDNA spanning exons 6-8 were subcloned and individual clones analysed. 77 individual clones revealed that 45% (34/77) of the transcripts were correctly spliced, while 55% (43/77) were aberrantly spliced. Furthermore, sequence analysis of the correctly spliced transcripts all harboured the p.A467T mutation suggesting that correct splicing of the c.1251-2A > T allele is completely impaired. In addition, these results also confirm that both alleles are approximately equally expressed and that indeed no NMD of the aberrantly spliced c.1251-2A > T allele occurs.

Molecular genetic testing of the patient's parents identified his father as a carrier of the novel c.1251-2A > T splice site mutation (Figure 1A and 1E) and his mother as a heterozygous carrier of the p.A467T mutation (Figure 1B and 1E).

**Figure 2:**
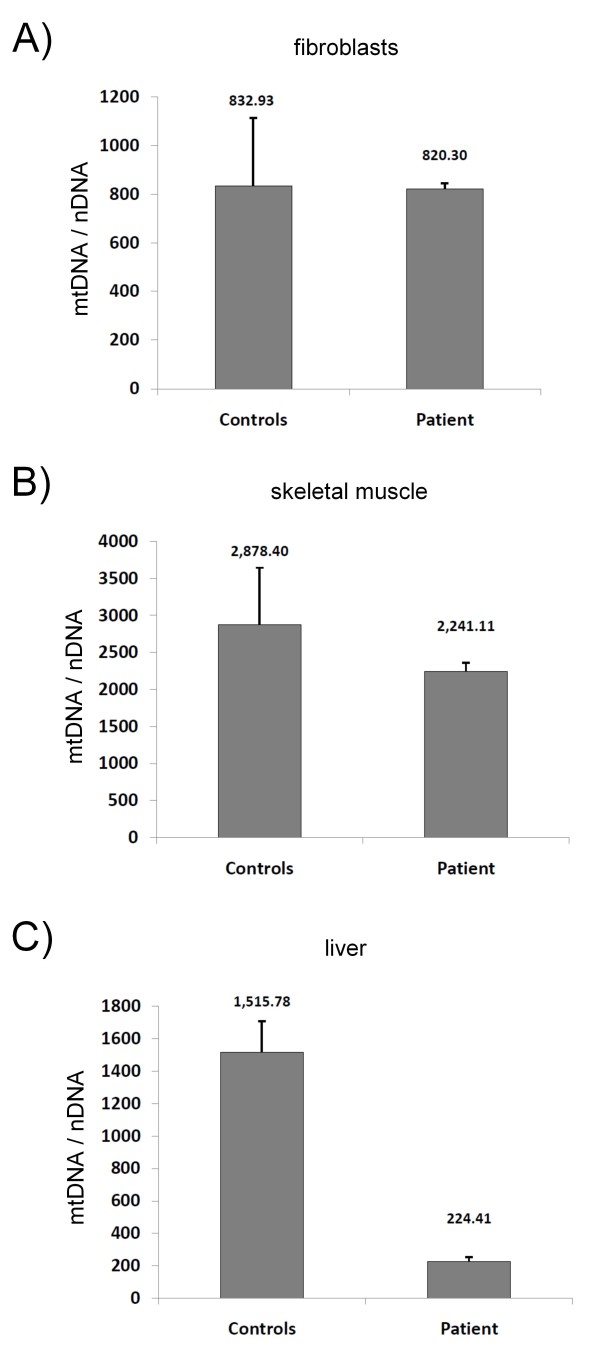
**Quantification of mtDNA**. Quantification of mtDNA depletion in muscle, liver and fibroblasts of the patient. mtDNA content was measured by qPCR and normalised to a nuclear gene (B2M). Control mean and +/-1SD: fibroblast 832 +/-280; skeletal muscle 2878 +/-766 and liver 1515 +/-192.

To further investigate the consequences of the *POLG *mutations, the mtDNA was quantitatively and qualitatively assessed using a combined qPCR approach. The analysis of DNA extracted from fibroblasts, liver and skeletal muscle revealed no deletions in the mtDNA in the tissues tested (results not shown). However, various degrees of mtDNA depletion were detected in the three tested tissues (Figure 2). In fibroblasts, the amount of mtDNA (820 molecules per cell) was insignificantly lower relative to the control mean (832 molecules per cell) (Figure 2A), whereas a mtDNA depletion of 22% was detected in muscle relative to the control mean (Figure 2B). The most pronounced mtDNA depletion was measured in the patient's liver tissue where 85% of the mtDNA was depleted relative to the control mean (Figure 2C).

**Figure 3:**
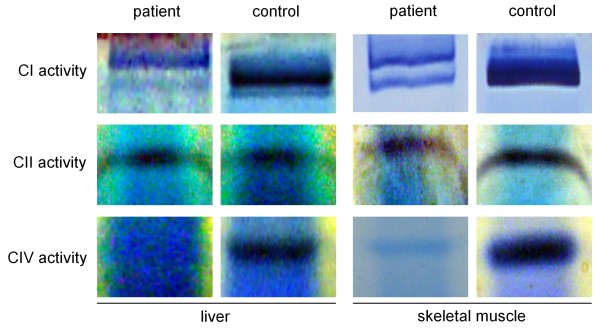
**Catalytic staining of OXPHOS-complexes**. Catalytic staining following separation of the OXPHOS-complexes by BN-PAGE showing decreased intensities of the bands corresponding to complex I and IV in patients liver and skeletal muscle. The bands corresponding to complex II are comparable to the control.

In order to assess the consequences of the mtDNA depletion on the OXPHOS activities, CI-V have been assayed. In the mitochondria from patient's fibroblasts, liver- and skeletal muscle tissues, OXPHOS enzymes (I, II, III, IV and V) were measured spectrophotometrically. In fibroblasts all activities were normal. The activities of complex I, III and IV were decreased in liver. In skeletal muscle, the activity of complex IV was decreased and the activities for complex I and II were in the lower control range (table 1). Catalytic staining in the BN-PAGE gel revealed a severe reduction of intensity for complex IV in liver and muscle (Figure 3). The intensities for complex I were also reduced in both tissues, but to a lesser extent. Staining for complex II was normal and comparable to the control (Figure 3).

**Table 1:** Patient's OXPHOS activities measured in three different tissues

	Fibroblasts		Skeletal muscle homogenate		Liver homogenate	
Enzymes	Patient	Controls (n = 22)	Patient	Controls (n = 26)	Patient	Controls (n = 12)
Complex I	0.29	0.19 - 0.46 (0.29 +/- 0.07)	0.16/0.13*	0.12 - 0.28 (0.19 +/- 0.04)	0.007/0.008*	0.22 - 0.76 (0.43 +/- 0.2)
Complex II	0.25	0.17 - 0.52 (0.33 +/- 0.09)	0.13/0.15*	0.14 - 0.36 (0.21 +/- 0.05)	0.68/0.75*	0.59 - 2.11 (1.35 +/- 0.45)
Complex III	0.42	0.35 - 0.87 (0.6 +/- 0.15)	0.74	0.55 - 1.11 (1.16 +/- 0.28)	0.26/0.3*	0.54 - 2.16 (1.47 +/- 0.49)
Complex IV	0.49	0.42 - 1.11 (0.75 +/- 0.18)	0.27/0.17*	0.57 - 1.77 (0.78 +/- 0.15)	0.35/0.42*	0.74 - 5.17 (2.1 +/- 1.2)
Complex V	0.16	0.14 - 0.42 (0.22 +/- 0.08)	0.19	0.19 - 0.65 (0.39 +/- 0.13)	0.42	0.25 - 1.14 (0.58 +/- 0.28)
Citrate synthase	172	106 - 317 (184 +/- 43)	124	70 - 169 (105 +/- 25)	68	21 - 40 (31 +/- 6.5)

Activities of the respiratory chain complexes are normalised to citrate synthase (CS) and are expressed as mU/mg mitochondrial protein. Mean value (+/- 1SD) of controls are indicated in brackets. Profound deficiencies below the range of controls are shown in bold. n = number of controls; * = measured as duplicate.

## Discussion

Mitochondrial depletion syndromes (MDS) are severe disorders often presenting themselves in early infancy or childhood. They comprise of a variety of features including profound weakness, encephalopathy, seizures and liver failure. A particular form of a hepatocerebral depletion is known as Alpers-Huttenlocher Syndrome (AHS) characterised by progressive neuronal degeneration in childhood, explosive onset of seizures, developmental delay, cortical blindness and spasticity followed by fulminant liver failure and parieto-occipital cerebral atrophy [29]. In AHS a depletion of the mtDNA is commonly observed, which is considered as a secondary phenomenon due to primary *POLG *mutations, which in turn leads to a defective system for oxidative phosphorylation (OXPHOS) [7]. However, *POLG *mutations in these phenotypes are not exclusive to the observed mtDNA damage.

Currently, there is no clear link between a particular *POLG *genotype and the resulting phenotype. However, with the characterisation of an increasing number of reported *POLG *mutations, patterns start to emerge. All AHS affected patients reported so far carry one of two linker mutations (p.A467T or p.W748S) in combination with either another linker mutation or a mutation located in the polymerase domain [19], whereas the p.A467T mutation is the most common mutation identified in *POLG*. It is present in all major POLG-related diseases: Alpers-Huttenlocher disease, ataxia-neuropathy syndromes and PEO.

Our patient showed a severe clinical phenotype and died due to valproate induced fatal acute liver failure. Analysis of the mtDNA content revealed a severe depletion in liver (approx. 90%), a less pronounced depletion in skeletal muscle (approx. 25%) and no depletion in fibroblasts. As a consequence, a combined respiratory chain defect involving complexes I, III and IV was measured in liver cells. In skeletal muscle, only complex IV showed a decreased activity suggesting that complex IV is the most vulnerable in mtDNA depletion syndromes. However, it is unknown which factors contribute to the tissue specificity of mitochondrial dysfunction in patients carrying POLG mutations. The finding of normal OXPHOS enzyme activities in our patient is also a common observation in other patients [16] and emphasises the need to investigate primary tissues as fibroblast analysis may give misleading results. The cellular mtDNA content may be an indicator of the underlying molecular mechanism linking genotype to phenotype and explaining the patient's acute liver failure.

Molecular genetic analysis of *POLG *revealed two linker region mutations, the c.1399G > A (p.A467T) and a novel splice site mutation c.1251-2A > T affecting the highly conserved splice acceptor site in intron 6. Analysis of the patient's parents confirmed that these mutations are present *in trans *in the patient. These findings are in good agreement with the observation that patients with two linker mutations exhibit a more severe clinical phenotype than patients carrying one linker and one polymerase domain mutation [30]. Furthermore, detection of the primary mutations in *POLG *did not only confirm the clinical diagnosis of Alpers syndrome, but also allowed a reliable prenatal diagnosis for the parents in the following pregnancy (Figure 1A and 1B).

Several inborn errors of metabolism are known to represent a risk factor for severe idiosyncratic reactions to VPA, including liver toxicity [31]. Many studies have focused on the interaction between VPA and mitochondrial function in general and mitochondrial disorders such as Alpers-Syndrome in particular, as conditions predisposing to severe VPA toxicity [32]. Recent studies gave evidence that *POLG *mutations can lead to a range of clinical phenotypes which predispose to the development of fatal liver failure after exposure to VPA [15,18]. Nevertheless, a single case report suggests that there may be mutations in the *POLG *gene associated with reversibility of the hepatotoxicity [33], The presented study extends the list of *POLG *mutations associated with VPA hepatotoxicity.

## Conclusion

Screening of *POLG *gene in mitochondrial diseases is helpful for confirming the diagnosis, especially in the case of AHS. *POLG *analysis offers the added benefits of carrier testing, prenatal diagnosis, postnatal pre-symptomatic diagnosis of siblings and optimised clinical management from the early stages of disease. Further this study contributes to the pathomechanism of *POLG *mutations and expands the knowledge of the genotype-phenotype correlation.

## Competing interests

This study has been funded by an unrestricted grant from Novartis. The authors have no conflict of interest with the source of funding of the study.

## Authors' contributions

AS participated in the design and coordination of the study, performed genetic and data analyses and drafted the manuscript. CBJ performed genetic and data analyses and participated in drafting the manuscript. DH and JMN performed biochemical and data analyses and participated in the study design and in drafting the manuscript. IK, CC and DCB performed the clinical investigations and participated in its design and in drafting the manuscript. SG participated in genetic data analyses and in the design and drafting the manuscript. All authors read and approved the final manuscript

## Pre-publication history

The pre-publication history for this paper can be accessed here:

http://www.biomedcentral.com/1471-2377/11/4/prepub

## Supplementary Material

Additional file 1 Primer sequences for PCR amplification of *POLG *exons Click here for file
