# Supplementary material for: Molecular and biochemical characterisation of a novel mutation in POLG associated with Alpers syndrome
Source: BMC Neurol. 2011 Jan 14;11:4. doi: 10.1186/1471-2377-11-4 (PMC3032677; doi:10.1186/1471-2377-11-4)
Supplement: Additional file 1 — Primer sequences for PCR amplification of POLG exons [file 1471-2377-11-4-S1.DOC]

**table - primer sequences for PCR amplification of *POLG*** exons

| exon no. | forward primer sequence (5’-3’) | reverse primer sequence (5’-3’) |
| --- | --- | --- |
| 2a | CAGTGTTGGGGACGCAGTAA | CCCTCCGAGGATAGCACTTG |
| 2b | GCAGCAACAGCAGCCTCA | TCCAGGTTGTCCCCGTAGAG |
| 2c | AGGTCGAGCACCTGCAGAA | ACACATCAGCGCTCCCTACG |
| 3 | TGTGCAGTGGTTGTTGTGGA | GCAAAGGCAACAGAGACACG |
| 4 | TTTGGTGTTGAAGGCTGTGTG | GCACCTCTGCCAGACTGTTG |
| 5 | GGCAGGAGCATAGTGCTTGG | ATCAGGTCCTGGCACAAGGT |
| 6 | TAGCTGCACTTGGGGAGATG | CCCAGATGGACACCACTGAA |
| 7 | TTTGAGCTGTGCCATGTCAGT | AGGCTAAGCCGAAGGCTAGG |
| 8 | GGATCTAGGCAGGGGACTGG | GTGGGGGAAGACAATCAGGA |
| 9 | TGTGATTGAGGGGGTCCTTC | GACTGAATGGCAGCAGGTCA |
| 10 | GGTGGGGACATTGTGAGAGA | TCCACTAGCCTGAGCTGACC |
| 11 | GTGGGCATCTGGTAATCAGC | CCTTCCCTGGGTGGAATACA |
| 12 | GACTGGCCTGCAGTGTCTGA | GGAACTCTGGCTGGGAAGAA |
| 13 | CAAGGAGAAAGCCTGGGTGA | TGCCTGAAATCACACTCTGTCC |
| 14 | CTGAGGTTCTGGGCTCAGTG | CATAGTCAGGCTGGCCCTCT |
| 15 | GGGCTCACCTTGAGTCCAGT | TGGGTCTTAGCAGGGTGCTT |
| 16 | GGTATGGTCTGCTGAGTGGTTG | GCCCTCAGAGCCCAGTTTCTA |
| 17 | GCAGTGCTGTCTGGCATTCT | GTCATCCACCCAAAGGCTGT |
| 18 | CTTGGAGACGCCCACTTTG | AGTTCAAGTAATGGGCAGGAGA |
| 19 | ACTAGCGTGGCACAGGAAGC | AGCAACCACCTCCCACTTCT |
| 20 | AGGATGGAGGGAGGGGTCT | GAAGGTGGGCAGAGGTGAAA |
| 21 | CCCTGTGGACCTTACCAATG | AAGGAACGCTCACCCAAAG |
| 22 | GAACAGATGGGGTGTTGGTC | AGCCTGAGTCAAGAGTGGATTC |
| 23 | ACATTACCGTTCGTGGCAAT | ACAATGCCCCTTGTCCTGTA |
